# Idiopathic Cervical Lymphocele Mimicking Congenital Lymphatic Malformation

**DOI:** 10.7759/cureus.99824

**Published:** 2025-12-22

**Authors:** Maaz Hamid, Muhammad Abdullah, Sabeeh Zahra, Meher Jehan

**Affiliations:** 1 Shifa College of Medicine, Shifa Tameer-E-Millat University Shifa College of Medicine, Islamabad, PAK; 2 General Surgery, Shifa International Hospital Islamabad, Islamabad, PAK

**Keywords:** bleomycin sclerotherapy, cervical lymphocele, cervical mass, lymphatic system, minimally invasive management

## Abstract

Lymphoceles are collections of lymphatic fluid that typically arise following surgical disruption of lymphatic channels, most commonly in the retroperitoneal or pelvic regions. Idiopathic presentations, especially in the cervical region, are extremely rare and may mimic congenital lymphatic malformations such as cystic hygroma and lymphangioma, complicating diagnosis. We present the case of a 30-year-old male with a neck mass that has been getting larger gradually and has caused no pain; it was associated with no previous trauma or surgery or history of infection. Imaging revealed a well-circumscribed, fluid-filled lesion that compressed adjacent cervical structures. Ultrasound-guided aspiration yielded clear lymphocyte-rich fluid, confirming the diagnosis of a lymphocele. The patient was successfully managed with bleomycin-based sclerotherapy, achieving complete resolution without recurrence over a five-year follow-up. This case emphasizes the importance of a comprehensive diagnostic approach, including clinical history, imaging, and cytological evaluation, to differentiate idiopathic lymphoceles from congenital lymphatic anomalies and highlights the efficacy of sclerotherapy as a minimally invasive therapeutic option.

## Introduction

Lymphoceles are fluid-filled collections that arise from the disruption of lymphatic channels, most commonly following surgical procedures. They are particularly associated with extraperitoneal surgeries and, less frequently, intraperitoneal procedures. The risk of lymphocele formation is notably increased after renal transplantation and lymphadenectomy for gynecologic malignancies. Additionally, vascular and spinal surgeries have been reported as contributing factors [1-5].

The clinical presentation of lymphoceles depends on their size and anatomical location. Many lymphoceles remain asymptomatic; however, larger collections may present with compressive features, including abdominal pain, fever, urinary frequency, deep venous thrombosis, and hydronephrosis [6-9]. Small lymphoceles often escape detection on clinical examination alone, necessitating diagnostic imaging modalities such as ultrasonography, computed tomography (CT), and magnetic resonance imaging (MRI) for accurate identification [10]. Fine-needle aspiration and biochemical analysis remain essential for confirming the diagnosis and differentiating lymphoceles from other cystic lesions [11].

Congenital lymphatic malformations, previously referred to as cystic hygromas, primarily develop in the neck, clavicular region, and axilla [12]. These malformations are characterized by multiloculated macroscopic cystic spaces of embryonic origin [13]. Similarly, lymphatic malformations, formerly termed lymphangiomas, predominantly affect the head and neck region in approximately 75% of cases [14]. These anomalies may present as isolated vascular malformations or in association with genetic syndromes such as Turner syndrome, Proteus syndrome, and CLOVES syndrome [15-17]. Both macrocystic and microcystic lymphatic malformations can clinically mimic lymphoceles, complicating diagnosis.

In contrast to congenital lymphatic malformations, lymphoceles are lymph-filled collections lacking an epithelial lining, most commonly located in the retroperitoneal space [18]. While small, spontaneous lymphoceles often remain asymptomatic, larger lesions may cause significant morbidity due to compression of adjacent structures, necessitating therapeutic intervention. Management strategies include fine-needle aspiration, sclerotherapy, and surgical excision [19-23].

Various treatment modalities have been explored for lymphatic malformations, including surgical excision, laser therapy, sclerotherapy, electrocoagulation, cryosurgery, and carbon dioxide laser therapy. These interventions aim either to remove the abnormal lymphatic tissue or to obliterate aberrant lymphatic channels [24]. Sclerotherapy, a widely used non-surgical approach, involves injection of detergent sclerosants, chemical irritants, or hyperosmolar agents to induce fibrosis and collapse of the anomalous lymphatic vessels [25].

The purpose of this clinical report is to highlight an unusual presentation of a cervical lymphocele of unexplained origin, discuss its diagnostic features, and review available therapeutic options. Through this documentation, we aim to contribute to the existing literature on lymphoceles and provide insights into optimal management strategies.

## Case presentation

A 30-year-old male presented to the surgical department with a complaint of a non-painful mass on the right side of the neck. The patient first noticed the mass approximately one year ago, but over the past four months, it had rapidly increased in size. He reported difficulty swallowing solid foods and persistent halitosis for the past three months. Additionally, he expressed cosmetic concerns and localized discomfort due to the mass effect. The patient denied any history of trauma or previous surgical procedures in the affected region. He also denied fever, arthralgia, or other systemic symptoms.

The patient was afebrile, with normal blood pressure and pulses. There was no discrepancy in blood pressure between both arms, and the right radial pulse was normal in rate, rhythm, volume, and character. No radio-radial delay was noted.

On examination, a well-defined, painless, firm, and fixed mass measuring approximately 7 × 6 cm was noted on the right side of the neck. The lesion extended craniocaudally from the level of the C5 vertebra superiorly to the T1 vertebra inferiorly, corresponding to the infrahyoid region with extension into the supraclavicular fossa. The mass exhibited a faint pulsation, suggestive of transmitted pulsations from the underlying subclavian artery. There was no overlying erythema, facial puffiness, or congestion. Neurological examination revealed mild weakness in head rotation to the left; however, no pain was elicited during movement. Upper limb motor and sensory functions were intact, with normal muscle tone and strength (5/5) bilaterally. Deep tendon reflexes, including biceps and triceps reflexes, were normal. There were no signs of axillary structure compression or venous drainage obstruction. No palpable lymph nodes were detected in the cervical or axillary regions.

**Figure 1:**
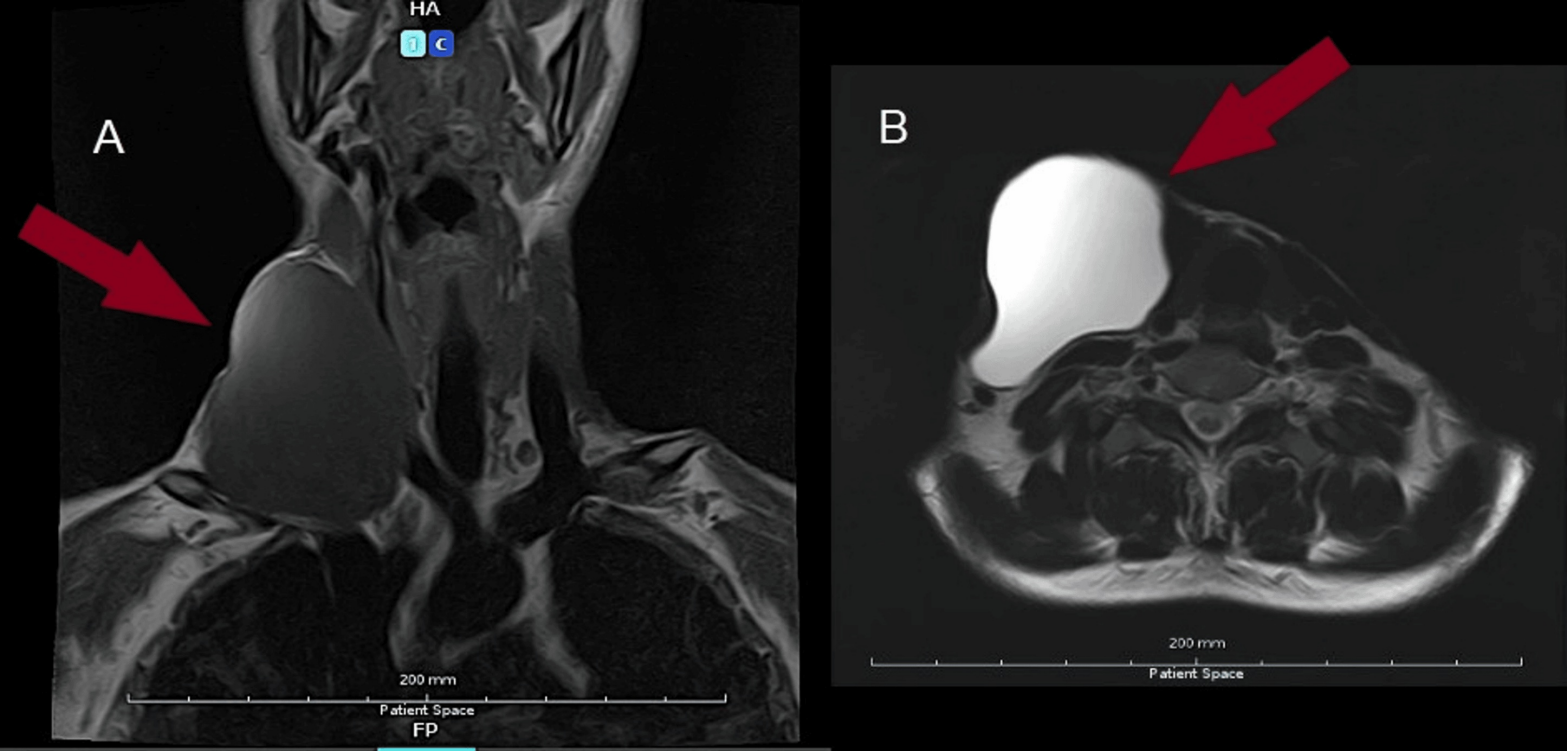
(A) MRI of the neck demonstrating a well-circumscribed, thin-walled, fluid-filled lesion with a lobulated architecture. The lesion shows no internal septations or enhancing solid components. It extends superiorly to the level of the C5 vertebra and inferiorly to the T1 vertebra. (B) MRI of the neck. No fistulous tracts or abnormal communications were identified MRI: magnetic resonance imaging

MRI of the neck without contrast (Figure 1) demonstrated a well-defined, thin-walled, fluid-containing lesion exhibiting a lobulated configuration. The lesion demonstrated no internal septations or enhancing solid components. It extended superiorly to the level of the C5 vertebra and inferiorly to the T1 vertebra. The lesion caused medial displacement and compression of the strap muscles. Anteriorly, it resulted in thinning and stretching of the right sternocleidomastoid muscle, with associated compression of the platysma. Medially, the lesion compressed the right lobe of the thyroid gland, leading to contralateral deviation and flattening of the gland, as well as displacement of the right lateral wall of the trachea. Despite these findings, the thyroid parenchyma appeared normal, and the tracheal lumen remained patent. The lesion extended into the right carotid space, resulting in splaying of the major cervical vessels and significant compression of the right internal jugular vein. However, flow voids were preserved. The internal carotid artery, external carotid artery, and common carotid artery on the right side were compressed and displaced, though without loss of normal flow signal. Inferiorly, the lesion extended into the supraclavicular region, compressing and posteriorly displacing the right subclavian artery. Posterior compression and flattening of the right anterior scalene muscle were also noted. Laterally, the lesion produced a contour bulge of the overlying skin. No fistulous tracts or abnormal communications were identified. Additionally, a dilated esophagus with evidence of stasis was observed. In light of these imaging results, the reporting radiologist diagnosed a lymphocele.

An ultrasound-guided aspiration was performed, yielding approximately 80 mL of clear yellow fluid. Despite residual fluid of 5 mL observed on ultrasound, the procedure was discontinued due to the increasing risk of iatrogenic injury, given the lesion’s close vicinity to the subclavian vessels, pleura, internal jugular vein, and thyroid capsule. Cytological interpretation of the aspirated sample revealed 100% normal lymphocytes (Table 1), reaffirming the diagnosis of a lymphocele.

**Table 1:** Fluid routine analysis and cytology TLC: total leukocyte count, RBC: red blood cells, LDH: lactate dehydrogenase

Parameters	Results	Units
Color	Yellow	-
Appearance	Turbid	-
Coagulum	Not present	-
TLC	64	/cumm
RBC	Positive	/cumm
Polymorph	20	%
Lymphocyte	80	%
Eosinophils	Nil	%
Monocyte	Nil	%
Protein	6.17	g/dl
Glucose	103	mg/dl
LDH	26	U/L
pH	8.0	-

Once the complete collapse of the cavity was achieved, sclerotherapy with bleomycin was performed. A total of 15 IU of sterile bleomycin was diluted in 12 mL of normal saline, yielding a concentration of 1.25 IU/mL. The bleomycin dose used was 0.5 mg/kg. The solution was retained within the cavity for at least 20 minutes before being aspirated. This procedure was repeated at weekly intervals as needed for up to three sessions.

Following each session, the patient was prescribed a three-day course of oral amoxicillin-clavulanate. Throughout the entire treatment period, the patient remained afebrile and exhibited no local signs of inflammation or significant discomfort. No recurrence of symptoms suggestive of lymphatic reaccumulation was observed during a five-year follow-up.

## Discussion

Lymphoceles are typically post-surgical collections of lymphatic fluid, often resulting from the disruption of afferent lymphatic vessels. They most commonly occur in the retroperitoneal or pelvic regions following surgeries such as renal transplantation, pelvic lymphadenectomy, and prostatectomy [2]. The fluid collects within a fibrous capsule devoid of epithelial lining, distinguishing lymphoceles from true cysts [26]. Though widely regarded as iatrogenic, idiopathic lymphoceles have been described in the literature, though rarely, with no identifiable preceding trauma, infection, or surgical intervention [27]. Our patient presented with a cervical lymphocele without any history of previous surgical or traumatic events, representing an uncommon idiopathic presentation.

Clinically, lymphoceles can range from asymptomatic incidental findings to symptomatic masses that compress adjacent structures [28]. Complications such as pain, fever, urinary frequency, venous thrombosis, hydronephrosis, wound infection, and delayed healing may occur in larger or infected lesions [6-9,29-30]. On physical examination, they may present as non-pulsatile, cystic masses, necessitating further imaging and diagnostic work-up.

Lymphoceles classically present as non-pulsatile masses on clinical examination. In this case, however, the lesion exhibited apparent pulsatility, attributable to transmitted pulsations from adjacent vascular structures due to its close anatomical proximity. Different techniques have been used to diagnose lymphocele, including both noninvasive and invasive methods. The usual tools that can be employed in evaluating the fluid are ultrasonography, CT, and MRI, followed by fluid aspiration and lab examination that will establish the presence of lymphatic fluid with a lymphocyte majority (more than 70%) on cell count, the low protein concentration (1.5 to 2 g/dL), and the low creatinine level [31]. Cytological studies of our patient were also consistent with the lab values mentioned above.

Ultrasonography reveals hypoechoic or anechoic oval-shaped structures [2], a thin-walled collection [32], and, sometimes, an internal septum and debris [19]. MRI will reveal thin-walled fluid collections with no contrast enhancement [32] and no visualization of increased flow, hemosiderin deposition, or hemorrhage [33]; nevertheless, these collections may occasionally contain blood products. CT will present a lymphocele as a non-enhanced, smooth, oval, thin-walled, or tubular structure after intravascular contrast administration [2,19,32,33].

The treatment option for a lymphocele depends on its location, size, and the structures involved. The modes of treatment are observation, needle aspiration, surgical resection, internal drainage by open or laparoscopic/thoracoscopic marsupialization, and percutaneous external drainage with or without sclerosing agents [2,19,23,11,34]. For infected lymphoceles, antibiotics alone are often sufficient [34].

Management includes ultrasound- or CT-guided needle aspiration to drain excess fluid, which treats pressure symptoms caused by lymphocele. Needle aspiration is first-line management for treating lymphocele. Still, data analysis also shows an increased risk of lymphocele recurrence of 80-90%, which leads to repeated needle aspiration, thus increasing the risk of infection to 25%-50% [2,11,23,34,35].

Percutaneous catheter drainage offers improved outcomes, with a reported success rate of 79-100% when maintained for approximately 14.5 days [11,20,23]. However, recurrence remains a concern (up to 63.6%) [20]. Adjunctive sclerotherapy, using agents such as tetracycline, doxycycline, bleomycin, ethanol, and povidone-iodine, induces fibrosis and obliteration of the lymphatic space, reducing recurrence [19,23,36]. The efficacy of sclerotherapy ranges from 79% to 100%, with resolution typically occurring within 9-36 days [23]. A study by Kerlan et al. [37] reported successful outcomes in four patients treated with bleomycin, none of whom experienced recurrence during an 11-month follow-up. Similarly, our patient was treated with bleomycin and demonstrated no evidence of recurrence during follow-up.

Surgical management, including laparoscopic or open marsupialization or peritoneal window formation, remains an option for refractory or complex cases, with a reported success rate of over 90%. However, it is associated with increased morbidity, longer hospital stays, and greater healthcare costs [23].

Differentiating lymphoceles from congenital lymphatic malformations is essential. Macrocystic lymphatic malformations (historically referred to as cystic hygromas) arise from a failure of embryologic communication between the lymphatic and venous systems and typically present in infancy or early childhood, most commonly in the cervical or axillary regions. However, delayed presentations in adulthood have been documented. Adult-onset macrocystic lymphatic malformations generally manifest as progressively enlarging supraclavicular masses. Imaging characteristically demonstrates a multilobulated cystic lesion, while histopathological examination reveals fibrous cyst walls with associated lymphoid cell infiltration [38].

Cervical lymphatic malformations (previously termed lymphangiomas), although congenital in origin, may occasionally present in adulthood, often precipitated by trauma, infection, or neoplastic processes. Radiographically and histologically, these lesions are characterized by dilated lymphatic channels lined by flattened endothelium and accompanied by lymphoid aggregates [39]. Clinically and radiographically, lymphatic malformations may closely mimic lymphoceles, resulting in diagnostic ambiguity. Therefore, a comprehensive diagnostic approach integrating clinical history, imaging studies, cytological evaluation, and histopathological analysis is essential to establish an accurate diagnosis and guide appropriate management.

## Conclusions

Idiopathic cervical lymphocele represents a rare clinical entity that may closely mimic congenital lymphatic malformations such as cystic hygroma and lymphangioma. This case highlights the diagnostic complexity and the importance of integrating detailed imaging and cytological analysis for accurate differentiation. Although needle aspiration provides symptomatic relief, adjunctive sclerotherapy significantly reduces the risk of recurrence and offers a favorable outcome with minimal invasiveness. Bleomycin-based sclerotherapy, as demonstrated in this case, can serve as a safe and effective alternative to surgical excision, particularly in anatomically sensitive regions. Early recognition and tailored management are essential to prevent complications and ensure long-term resolution. Future studies should focus on multicenter evaluations of the long-term efficacy and safety of sclerosing agents, as well as comparative analyses between sclerotherapy and surgical excision to establish standardized treatment protocols.
